# Co-design of a personalised digital intervention to improve vegetable intake in adults living in Australian rural communities

**DOI:** 10.1186/s12889-024-17641-8

**Published:** 2024-01-10

**Authors:** Katherine Mary Livingstone, Jonathan C Rawstorn, Laura Alston, Stephanie R Partridge, Amber Bastian, Kate Dullaghan, Sarah A McNaughton, Gilly A Hendrie, Lauren C Blekkenhorst, Ralph Maddison, Yuxin Zhang, Scott Barnett, John C Mathers, Stephanie L Godrich

**Affiliations:** 1 Institute for Physical Activity and Nutrition (IPAN), School of Exercise and Nutrition Sciences, Deakin University, 3220 Geelong, Victoria Australia; 2 Deakin Rural Health, School of Medicine, Faculty of Health, Deakin University, Geelong, Australia; 3 Engagement and Co-Design Research Hub, School of Health Sciences, Faculty of Medicine and Health, The University of Sydney, Sydney, NSW Australia; 4 Human Health Program, Health & Biosecurity, CSIRO, 5000 Adelaide, SA Australia; 5 Nutrition and Health Innovation Research Institute, School of Medical and Health Sciences, Edith Cowan University, Perth, Australia; 6 Medical School, Royal Perth Hospital Unit, The University of Western Australia, Perth, Australia; 7 Applied Artificial Intelligence Institute (A²I²), Deakin University, Geelong, Australia; 8 Human Nutrition Research Centre, Centre for Healthier Lives, Population Health Sciences Institute, Newcastle University, NE2 4HH Newcastle upon Tyne, UK; 9 School of Medical and Health Sciences, Edith Cowan University, 6230 Bunbury, WA Australia; 10 Institute for Physical Activity and Nutrition, School of Exercise and Nutrition Sciences, Deakin University, Melbourne Burwood Campus, 221 Burwood Highway, 3125 Melbourne, Victoria Australia

**Keywords:** Co-design, Workshop, Digital health, Behaviour change, Intervention, Vegetable intake, Adults

## Abstract

**Background:**

Diets low in vegetables are a main contributor to the health burden experienced by Australians living in rural communities. Given the ubiquity of smartphones and access to the Internet, digital interventions may offer an accessible delivery model for a dietary intervention in rural communities. However, no digital interventions to address low vegetable intake have been co-designed with adults living in rural areas. This paper describes the co-design of a digital intervention to improve vegetable intake with rural community members and research partners.

**Methods:**

Active participants in the co-design process were adults ≥ 18 years living in three rural Australian communities (total *n* = 57) and research partners (*n* = 4) representing three local rural governments and one peak non-government health organisation. An iterative co-design process was undertaken to understand the needs (pre-design phase) and ideas (generative phase) of the target population. Eight online workshops and a community survey were conducted between July and December 2021. The MoSCoW prioritisation method was used to help participants identify the ‘Must-have, Should-have, Could-have, and Won’t-have or will not have right now’ features and functions of the digital intervention. Workshops were transcribed and inductively analysed using NVivo. Convergent and divergent themes were identified between the workshops and community survey to identify how to implement the digital intervention in the community.

**Results:**

Consensus was reached on a concept for a digital intervention that addressed individual and food environment barriers to vegetable intake, specific to rural communities. Implementation recommendations centred on (i) food literacy approaches to improve skills via access to vegetable-rich recipes and healthy eating resources, (ii) access to personalisation options and behaviour change support, and (iii) improving the community food environment by providing information on and access to local food initiatives.

**Conclusions:**

Rural-dwelling adults expressed preferences for personalised intervention features that can enhance food literacy and engagement with community food environments. This research will inform the development of the prototyping (evaluation phase) and feasibility testing (post-design phase) of this intervention.

**Supplementary Information:**

The online version contains supplementary material available at 10.1186/s12889-024-17641-8.

## Background

Australians living in rural communities are more likely to experience higher rates of preventable disease than those living in major cities [1]. Compared to major cities, adults living in rural communities visit their general practitioners less often and have reduced access to nutrition professionals– mostly due to health care workforce challenges and geographical barriers [2]. Modifiable risk factors for chronic diseases, such as high blood pressure and suboptimal diets, are increasing the burden on rural health care systems [3].

Diets low in vegetables are a main contributor to the health burden experienced by rural Australians [4], which is exacerbated by socioeconomic inequities [5, 6]. While vegetable intake is slightly higher in rural areas compared with major cities, only 11% of adults living in rural areas meet recommended intakes [7]. High quality interventions can effectively change dietary intake, however sustaining change over time is more difficult to achieve. Further innovation in intervention design is needed to have a greater impact on dietary intake and risk of chronic disease [8].

Digital interventions cover a range of technologies that can be used to collect and share a person’s health information [9]. Delivery modes include a broad scope, such as smartphone applications, websites and mobile text messaging, and provide an accessible delivery model for most adults [10]. As a result, digital interventions can be innovated with new functionalities, such as personalisation to meet the needs of communities, including those in rural areas [11, 12]. However, digital interventions encounter the persistent challenge of low retention, limiting their potential to improve diet and health outcomes [11, 12].

Low retention with digital interventions can be addressed by using collaborative, or co-design, research methods [13]. Co-design practices embed stakeholders in the intervention creation and evaluation process, thereby helping to ensure that interventions are person-centred and more acceptable to the target population [13, 14]. The Sanders and Stappers [15] model of co-design outlines four phases: pre-design, generative, evaluative and post-design. The pre-design phase aims to understand the lived experiences of the target population, the generative phase guides the target population to produce ideas and concepts that can be designed and refined in an iterative process, and the evaluative phase involves prototype testing and refining with the target population [15]. Despite the advantages of co-design methods [14], our recent systematic review of digital interventions to increase vegetable intake identified that co-design practices were under-utilised. Moreover, no digital interventions had been co-designed with adults living in rural communities [16]. Therefore, this paper describes the co-design of a personalised digital vegetable intervention with adults living in rural communities and local government and non-government research partners.

## Methods

The study received ethics approval from the Deakin University Human Ethics Advisory Group - Health (HEAG-H 83_2021) and has been reported according to the Consolidated Criteria for Reporting Qualitative Research (COREQ) checklist (Supplemental Table 1) [17].

### Study design and participants

**Fig. 1:**
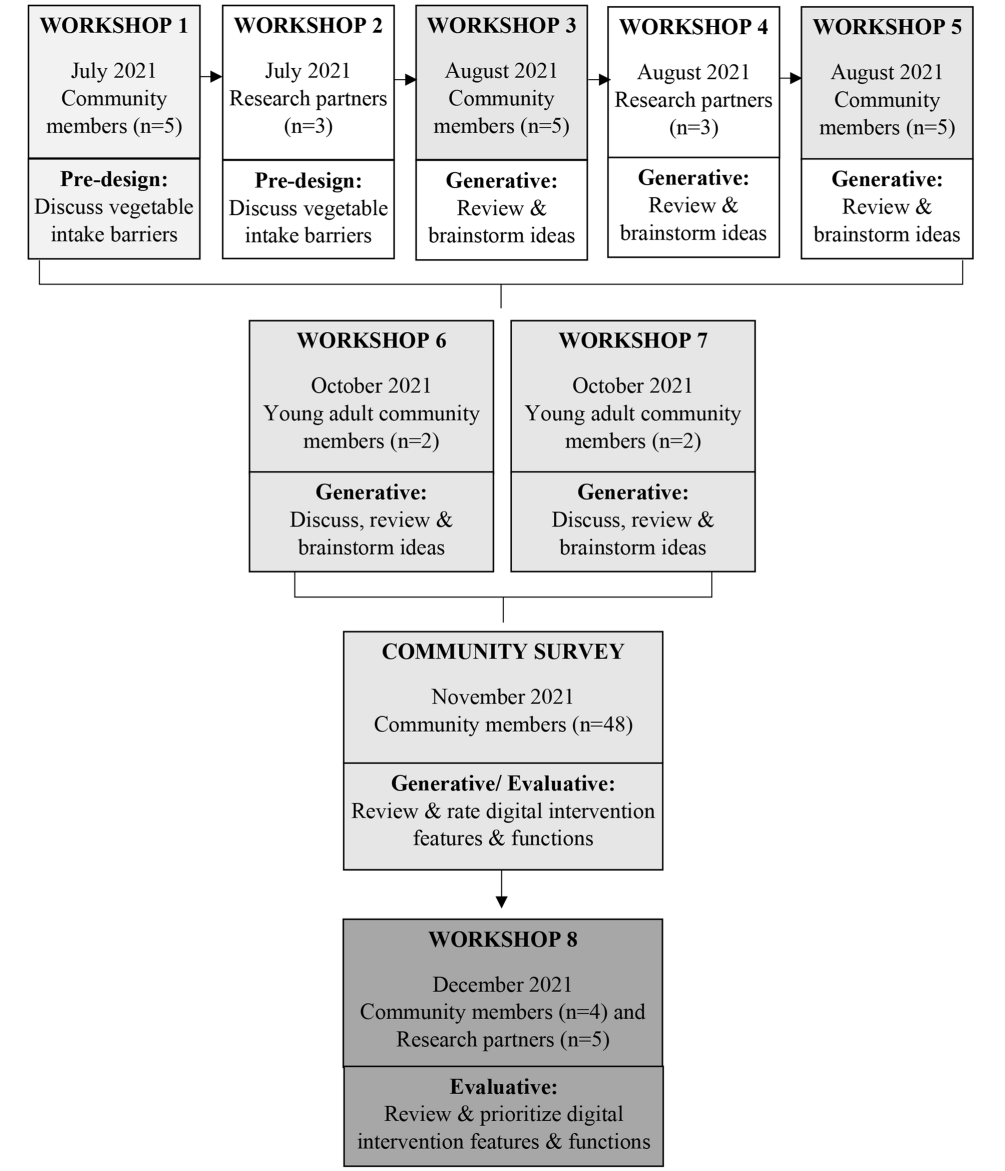
Flow diagram of iterative co-design workshops and community survey with community members and research partners. White boxes represent research partner workshops; light grey boxes present community member (adults) workshops/survey; dark grey box represents research partners and community members together in the same workshop. The same community members attended workshop 1–5. Different community members attended workshops 6 and 7. Community members from workshops 1–7 were invited to join workshop 8. Workshops reflect the co-design phases of pre-design, generative and evaluative as defined by Sanders and Stappers [14]

This study utilised a mixed-methods approach, guided by co-design (participatory) and intervention mapping frameworks [15, 18]. A total of eight interactive co-design workshops and an online community survey were conducted between July 2021 and December 2021 (Fig. 1). Active participants in the co-design process were adults living in the target communities (referred to as community members) who met defined eligibility criteria, and research partners. Research partners represented three rural local governments (food systems, environmental and health officers) and one peak non-government national health organisation. Research partners were selected based on established connections with the research team and ability to achieve rural and national reach. Eligibility criteria for community members included adults aged ≥ 18 years, consuming less than 5 serves of vegetables daily, ownership of an Android or iOS mobile device, internet connection, and English as the primary language spoken at home. Eligibility was also determined based on whether participants lived in the Australian rural communities of Loddon Campaspe in Victoria or the South-West Region of Western Australia. These regions are classified as regional centres (MM2: inner or outer regional areas with, or within 20 km road distance of, a population > 50,000) and large (MM3: inner or outer regional areas with, or within 15 km road distance of, a population of 15,000 to 50,000), medium (MM4: inner or outer regional areas with, or within 10 km road distance of, a population of 5,000 to 15,000) and small (MM5: all other inner or outer regional areas) rural towns based on the Modified Monash Model remoteness categories [19], where each remoteness category reflects comparable socio-economic profiles [19].

### Sampling and recruitment

#### Workshops

Convenience and purposeful sampling were used to recruit adults living in the target communities. Consistent with best practice guidelines for qualitative research [20, 21], and to include members from each community, the target sample size for the workshops was 14. Paid social media advertising, targeted based on location, age, and sex, were used on Facebook and Instagram between June and July 2021. Individuals (community members) completed online screening questions in Qualtrics Survey Software (Qualtrics XM) to determine eligibility to participate in a brief online screening survey and three virtual workshops (workshop 1; workshop 3; workshop 5). If they met the eligibility criteria and provided online consent to participate in the study, they were prompted to complete a brief online screening survey that collected information on demographic characteristics (age; education; postcode), health and eating behaviours (household food shopping; household food preparation; smoking; self-reported health status), vegetable intake (serves/day) based on a validated self-report question [22], and digital device use (hours/day). Participants were then contacted by a member of the research team (AB) to arrange workshop attendance. Participants who attended all three workshops were compensated with an AUD60 e-gift voucher for their time. Participants also provided consent to be invited to attend a final workshop that included community members and research partners (workshop 8). All participants who attended workshop 8 received an additional AUD50 e-gift voucher. Research partners provided written consent to participate in the workshops (workshop 2; workshop 4; workshop 8). Research partners did not receive compensation for their time due to mutually agreed in-kind time commitment to the project at the study commencement.

A second round of workshops (workshop 6 and workshop 7) were conducted to capture the views of young adults (aged 18–35 years), who were under-represented in the first round of workshops. Two separate workshops were necessary to accommodate the availability of participants. Additional social media advertisement (paid and targeted) on Facebook and Instagram were undertaken in September and October 2021 to specifically recruit young adults into workshop 6 and workshop 7. The same processes were untaken as described above, except eligible adults were those aged 18–35 years of age.

#### Community survey

Adults were eligible if they met the predefined eligibility criteria. Convenience and purposeful sampling were used to recruit participants over a two-week period during November 2021, using paid and targeted advertising on social media (Facebook and Instagram). To capture a wider range of perspectives than the workshops, the target sample size for the survey was doubled to ≥ 30 participants. Interested individuals clicked on a link to access the open online survey, which was delivered via Qualtrics Survey Software (Qualtrics XM). If individuals met the eligibility criteria and provided online consent, they completed the same screening questions as the workshop participants (i.e., demographic characteristics, health and eating behaviours, readiness for behaviour change [23] and digital device use) as well as 21 questions on the digital intervention features and functions. Closed questions were used to rate features and functions using four-point Likert scales. For example, “What features of recipes would you use?” included a list of 15 functions that could be rated from “I would use this often” to “I would never use this”. Open ended questions were used to collect information on reasons for rating. Except for screening questions, all items were voluntary and used adaptive questioning where applicable. Participants could opt in for the chance to win a AUD50 e-gift voucher as compensation for their time. Selection of the winning participant was conducted randomly using Microsoft Excel (Microsoft 365 MSO).

### Workshops 1–7

The first round of workshops (1–5) was conducted with community members (1, 3 and 5) and research partners (2 and 4) during July and August 2021. The second round of workshops (6 and 7) was conducted with community members in October 2021. Each workshop was 90 min in duration. To comply with COVID-19 restrictions and bridge geographic distances between Victoria and Western Australia, the workshops were conducted and recorded online via Zoom software (Zoom Video Communications Inc., San Jose, CA, USA). The online interactive platforms Padlet (Padlet, San Francisco, CA, USA) and Miro (Miro, San Francisco, CA, USA) were used to share ideas and record participant responses. The same researcher (AB) facilitated all workshops, a female research fellow (PhD) with experience conducting qualitative research [24] who was not known to the participants prior to this research. Only this researcher and the study participants were present in the workshops.

The workshops followed a staged co-design process used previously in community settings, [25] where the workshops aimed to (i) sensitise participants to the topic and then (ii) facilitate creativity and ideation to focus on new ideas (pre-design and generative phases of co-design).

Supplemental Table 2 provides details of the content of each workshop. Briefly, workshops 1 (community members) and 2 (research partners) encouraged participants to discuss barriers to vegetable intake and brainstorm ideas to overcome them in a digital intervention. After initial brainstorming, three exemplars from existing digital tools were provided to give ideas of what other tools have included, i.e., recipes; goal-setting; food sharing. These were selected by the researchers to cover potential barriers identified in the literature [16]. Participants were encouraged to reflect on whether they liked the exemplars or not, and why. The same community members attended workshops 1, 3 and 5 so that ideas and concepts from previous workshops could be built on, and findings from research partner workshops could be integrated. The same research partners attended workshops 2 and 4, which incorporated ideas and concepts from the community workshops. Ideas from workshops 1–5 were summarised in workshops 6 and 7 for discussion and input of new ideas from the perspective of younger adults. This iterative workshop design aimed to collect and integrate ideas from the community members and research partners and to avoid any perceived power imbalances that may have arisen by including both groups in the workshops together.

### Community survey

Ideas and concepts from workshops 1–7 were integrated into an online community survey to get broader feedback on the intervention design (generative and evaluative phases of co-design). This survey included a total of 21 questions that covered co-designed functions of the digital intervention. Participants were asked to indicate how often they would use certain functions, with sub-questions to gather additional details about function preferences.

### Workshop 8

Ideas and concepts from workshops 1–7 and the community survey were presented to community members and research partners together in a final 90-minute co-design workshop (workshop 8) conducted in December 2021. Similar to workshops 1–7, the final workshop was conducted and recorded using Zoom software and utilised the online platforms Padlet and Miro to share ideas and record responses. The workshop utilised the MoSCoW prioritisation method to reach a consensus on the features of the digital intervention (evaluate phase of co-design). This approach asks participants to prioritise which features should be included in the intervention using the following four categories: Must-have (nonnegotiable and essential to the function of the digital intervention); Should-have (important and will add significant value, but not vital); Could-have (nice to have but will only have a small impact if left out) and Won’t have, or maybe later (not a priority but could be added as a function at a later stage).

### Data analysis

Workshop recordings were transcribed verbatim and any identifying information removed. Data from any participants who withdrew prior to analysis were not included. The workshop transcripts were inductively analysed by two researchers (AB and SG) in QSR NVivo (Lumivero, Denver, CO, USA) [26]. Content analysis was used to understand and synthesise participants’ ideas about the intervention. Data from the online consumer survey, the co-design community workshop and partner workshop were triangulated to identify convergent and divergent themes: Content from each data source was examined in the theme development and points of interest relating to each theme were described to provide three perspectives on each theme. Data familiarisation (reviewing workshop transcripts) occurred initially, and a coding framework devised and expanded as analyses progressed. Both researchers (AB and SG) agreed on the initial coding framework. One team member (AB) coded the data, and both researchers reviewed, refined, and synthesised the themes. This systematic analysis of the data was conducted (i) against each theme/parent node, (ii) across subthemes/child nodes and (iii) across the entire data set to represent the participants’ ideas. From these themes, implementation recommendations were generated through discussion between researchers and reflected examples of potential implementation pathways in this setting rather than an exhaustive list. Each theme was mapped to the NOURISHING framework to identify alignment of each theme with policy actions related to the three domains of behaviour change communications, the food environment and the food system [27]. Example quotes from participants in the workshops were selected and added to the coding framework document to reflect each theme.

Quantitative data from the consumer survey were analysed using Stata (Version SE 15.0; StataCorp LLC, USA). Postcodes were used to assign an Index of Relative Socio-economic Disadvantage, which summarises information about the economic and social conditions of people and households within an area [28]. Descriptive statistics were reported using mean (standard deviation) for continuous variables and frequency counts for categorical variables.

## Results

### Participants

#### Workshops

For the first series of community member workshops (workshops 1, 3, 5), a total of 51 individuals consented to participate in the workshops and 19 provided complete responses. Of these, eight participants indicated they were willing and able to attend all three community member workshops. Two participants withdrew prior to the workshop commencement, and one withdrew after the first workshop. Five community member participants completed workshops 1, 3 and 5 and three research partners completed workshops 2 and 4 (Fig. 1).

For the second series of community member workshops targeting young adult community members (workshops 6 and 7), a total of 45 individuals provided consent, and eight participants indicated that they were willing and able to attend a workshop. To accommodate participant availability, two workshops (workshops 6–7) were delivered. Four participants withdrew prior to the workshop, leaving two males attending workshop 6 and two females attending workshop 7 (Fig. 1).

In total, nine community members participated in workshops 1–7 (Table 1). The majority (78%) of participants were female (n = 7) and living in Loddon Campaspe (n = 7), with an average age of 44 years (range 23–62 years). At least 50% of participants came from disadvantaged areas within these locations. On average, participants reported consuming 1.4 (SD 0.96) serves of vegetables per day (3.6 serves lower on average than recommended intakes). More than three quarters of participants (78%) were responsible for household food shopping and preparation.

**Table 1:** Participant characteristics of community members from workshops and community survey

Characteristics	Workshops 1–7 (total)	Individual workshops			Community survey
		Workshops 1,3,5 (community members)	Workshop 6 (young males)	Workshop 7 (young females)	
N	9	5	2	2	48
Female, n (%)	7 (78)	5 (100)	0 (0)	2 (100)	39 (81)
Age, (years), mean (SD)	44 (13.7)	55 (5.17)	25 (2)	34 (0.50)	43 (13.8)
Education, n (%)^1^					
Low	1 (11)	1 (20)	0	0	2 (4)
Middle	3 (33)	2 (40)	1 (50)	0	4 (8)
High	5 (56)	2 (40)	1 (50)	2 (100)	42 (88)
SEIFA, n (%)^2^					
Most disadvantaged area	2 (22)	2 (40)	0	0	10 (21)
Least disadvantaged area	7 (78)	3 (60)	2 (100)	2 (100)	37 (79)
Vegetable intake (serves/day), mean (SD)	1.4 (0.96)	1.2 (0.75)	1.5 (0.50)	2.5 (0.50)	2.54 (1.03)
Does household food shopping, n (%)					
Yes, usually	7 (78)	5 (100)	0	2 (100)	42 (88)
Sometimes	2 (22)	0	2 (100)	0	5 (10)
No	0	0	0	0	1 (2)
Does household food preparation, n (%)					
Yes, usually	7 (78)	4 (80)	1 (50)	2 (100)	36 (75)
Sometimes	2 (22)	1 (20)	1 (50)	0	10 (21)
No	0	0	0	0	2 (4)
Behaviour change stage, n (%)					
Contemplation	4 (44)	2 (40)	2 (100)	0	1 (2)
Preparation	1 (11)	1 (20)	0	0	6 (12.5)
Action	2 (22)	1 (20)	0	1 (50)	23 (48)
Maintenance	2 (22)	1 (20)	0	1 (50)	18 (37.5)
Smoking, n (%)					
Never/former	7 (78)	5 (100)	1 (50)	1 (50)	45 (94)
Currently smoke	2 (22)		1 (50)	1 (50)	3 (6)
Health, n (%)					
Fair/good	6 (67)	3 (60)	2 (100)	1 (50)	19 (40)
Excellent/very good	3 (33)	2 (40)	0	1 (50)	29 (60)
Mobile devise use (hours/day), mean (SD)	3.3 (2.87)	2.2 (1.32)	7.5 (2.50)	2 (2.00)	3.59 (2.06)

SEIFA, Socio-Economic Indexes for Areas

^1^High = Higher University degree (e.g., Graduate Diploma, Masters, PhD); Certificate/diploma (e.g. childcare, technician), Middle = Trade/apprenticeship (e.g. hairdresser, chef); Year 12 or equivalent (e.g. Higher School Certificate), Low = Year 10 or equivalent

^2^Most disadvantaged = 1–3 SEIFA, Least disadvantaged = 4–10 SEIFA

#### Community survey

A total of 106 individuals consented to participate in the community survey. Of these, 48 individuals met the inclusion criteria and provided complete survey responses. Most participants (81%) were female, most were from Loddon Campaspe (81%), with an average age of 43 years. On average, participants reported consuming 2.5 (SD 1.0) serves of vegetables per day (Table 1), which was 1.1 serves higher than that of workshop participants.

#### Final workshop

Four community participants (a subset of the participants from workshops 1–7) and five research partners attended the final workshop. Three of the four community participants lived in Loddon Campaspe. Two of the research partners had participated in the previous workshops, however, due to changes in staffing, three were new to the project.

### Workshop themes

Four overarching themes for intervention features were identified from workshops 1–7: recipes; social and community; personalisation; and cooking resources (Table 2). Co-design was also mentioned by participants as a strength of the study. These themes are listed in order of discussion frequency.

**Table 2:** Overarching themes and subthemes arising from workshops 1–7

Overarching theme	Sub theme	Description of sub theme
Recipes	Family-friendly	Having recipes that are suitable for the whole family, or can be tweaked to suit both adults and children
	Colourful photos	Including colourful and appealing photos of food that encourages people to try the recipe
	Commonly used quantities	Using measures such as ‘handful’ and ‘pinch’ rather than grams, so that equipment is not needed to measure ingredients
	Downloadable and printable	Being able to download/save recipe and /or print the recipe out
	Export ingredients to a list	Being able to export ingredients from recipes into a shopping list
	Feature vegetables in season	Having a focus on/including recipes for vegetables currently in season
	Meal planning	Choose recipes and get assistance with planning meals for the coming days/week
	Indicate cost	Have information on the approximate cost for ingredients for the recipe
	Frequently Asked Questions (FAQs)	Include facts and FAQs on vegetables to help overcome preconceptions
	Preparation and cooking time	Indicate the amount of time it will take to prepare and cook the recipe
	Search for other recipes	Search for recipes by typing in which vegetables you have
	Swap vegetables for those in season / another vegetable	Being able to swap vegetables for those in season, for vegetables already in the fridge, or due to allergies or family food preferences
	Tips	Include tips with the recipes on food preparation, cooking or storage
Social and community	Cooking events (in person)	Having local cooking classes or events in the community that people can attend in person
	Food banks	Make people more aware of food banks available
	Food growing, sharing or U pick	Linking into local food growing, food sharing or ‘U pick’ initiatives
	Link ingredients to local producers	Connect recipes with locally grown vegetables and what is sold at local farmers markets
	Work with local food retailers	Connect with local supermarkets and green grocers to promote vegetable intake
	Local markets	Supporting local markets to promote vegetable intake
	Upcoming local events	Promoting local food events or linking vegetables into local events
	Links with community groups	Linking into existing groups and services within the community e.g. Men’s shed, libraries, community centres
	Chat function	Having a platform where people can chat with other members about things
	Multi-pronged approach	Use a variety of different strategies/activities to engage the community
	Promote through social media or media	Use social media or media (e.g. local radio, television, newspapers) to promote the digital intervention in the local community
	Promote community grants	Promoting grants that are available to local communities to encourage vegetable intake
Personalisation	Adapt to different family sizes	Adapt recipes to suit different families i.e. cooks enough for 2, 4 or 5 people
	Key word search	Being able to search for recipes, or local initiatives using key words
	Kids’ section	Have a kids’ section where they can play interactive games focused on vegetables
	Save pages of interest	Being able to save recipes or pages of interest to go back to later
	Set goals and challenges	Being able to set goals and challenges either individually, or with a group of family or friends
	Tailor content to local area	Being able to search for local community events, such as farmers’ markets, based on location
	Text messages or push notifications	Being sent text messages or notifications to be reminded about something (such as a goal), or alerted to new content on the digital intervention
Cooking resources	Demonstrate food storage, preparation, or cooking	Provide information on cooking and food preparation skills, including tips
	Feature local chefs or community members	Use local chefs or community members in cooking videos
	Show children cooking or include child-friendly options	Show children cooking and preparing vegetables in cooking videos and/or include child-friendly options

#### Recipes

Recipes were the most discussed feature of the digital intervention. Participants requested that the proposed digital intervention include child- or family-friendly recipes, colourful photos, vegetables in season, and commonly used quantities (such as a pinch or handful) to remove barriers for people who do not have measuring equipment at home, i.e.:

Not everybody has the equipment. I mean… they’re going 60 g or something of baby spinach. Well, then you’ve got to try and work all that out, and so that’s more cost, it’s not just going there and having a scale. Not everybody’s got… scales at home and you certainly can’t use your body scales to weigh it. (Workshop 3, female community member aged 58, Loddon Campaspe Victoria)

Participants suggested that recipes be downloadable and printable and that they indicate the expected preparation and/or cooking time. Participants also suggested that the digital intervention should have the functionality to export recipe ingredients to a shopping list, and use recipes for meal planning, as well as a tips section. Furthermore, participants wanted the digital intervention to include a recipe search for specific vegetables, i.e.:

Sometimes if you’ve got a certain vegetable left or a certain couple of vegetables left, a quick Google search or go to a particular website for those couple of vegetables to come up with recipes. (Workshop 2, research partner)

#### Social and community

A social and community component of the digital intervention was discussed in all workshops. Participants wanted the digital intervention to include information on local community events and initiatives such as markets, food sharing or growing, in-person cooking events and access to food banks and local producers. There was also discussion around a chat function to facilitate linking-in with other members of the community.

The range of supports required to ensure uptake and engagement with a digital intervention were raised in all workshops. This included linking-in with local community groups and food retailers, and having a section offering local grants to fund community food sharing initiatives. There was also discussion around how a variety of different strategies might be needed depending on the target audience, such as using both social and traditional media (i.e., local radio, television, newspapers) to promote the digital intervention in the community. A combination of online and in-person community engagement events were deemed important, i.e.:

Cast a wider net to draw in more people who normally do not look for these things through other means of communication such as radio, tv and food retailers (Workshop 6, male community member aged 23, South West region of Western Australia).

#### Personalisation

Personalisation of the digital intervention was frequently discussed. For example, the ability to save pages of interest and tailor content to the local area were considered important features. Being able to adapt recipes to different family sizes was also a suggested feature. An interactive section was discussed, with individual or family/friends goal-setting, and challenges with push notifications. There was a variety of perceptions on the value of these features, i.e.:

I think probably there’s a proportion of people who find that quite appealing, who like… monitoring things. Depending on people’s situations. They may not always have their phones on them during the day. Some workplaces don’t really allow that. So they wouldn’t necessarily be seeing throughout the day the information. I know personally, I am always looking at ways to use my phone less, so I think it’d be someone who’s really motivated to have their phone on them constantly and be checking it. (Workshop 2, research partner)

#### Cooking resources

Cooking resources, such as videos, were discussed. They received good support with participants suggesting these would be a good avenue to demonstrate basic cooking and food preparation skills. There was support for using local chefs or community members, to ‘localise’ the digital intervention, however, discussion around the practicality of implementing this across different geographical areas questioned the inclusion from a practical perspective. There was also good support for showing children cooking in the videos, to encourage children to be involved in cooking in the home, i.e.:

And also from the perspective of my kids I don’t think it necessarily needs to be a well-known chef or anything doing it. I think they would just really like to see kids cooking in the kitchen– would excite them and be like, “Hey, I could try that.” Almost like Junior Master Chef where a kid cooks something and then they’re like, “Oh, I wanna try and cook it like that kid did.” They just tend to like watching kids on YouTube doing stuff and then trying to copy them. (Workshop 7, female community member, aged 33, South West region of Western Australia).

#### Co-design

Participants discussed how they had enjoyed the experience of the co-design workshops and valued having their ideas and thoughts incorporated, i.e.:

And for me, the same, getting the opportunity to participate and say what can work for us, ‘cause sometimes you get like the little handouts and magazines and stuff and you look at them and you’re like, “Oh, man, that’s not gonna work for us,” and then you don’t glance at it, whereas now, just being able to be involved in it and then see how it evolves would be really exciting. (Workshop 7, female community member aged 33, South West region of Western Australia)

I’ve actually personally enjoyed being able to give you my views on what would be helpful. (Workshop 7, female community member aged 34, Loddon Campaspe Victoria)

### Common and divergent themes between the workshops and the community survey

Triangulation of workshop and community survey themes identified convergent and divergent themes for which features should be included in the digital intervention.

#### Convergent themes

An intervention that was a resource for quick and easy recipes formed a large part of the discussions in the community and research partner workshops and was supported by the community survey. The most popular features of recipes supported by the community survey was being able to swap vegetables in recipes for those in season, and a key word search function to be able to find or filter recipes. Furthermore, featuring vegetables in season, and having a way to plan meals for the week and create shopping lists, were strongly supported in the workshops and the community survey. Family-friendly recipes were also well supported in both the workshops and community survey. There was a strong desire to have the ability to personalise the digital intervention content based on individual needs. For example, a page-saving function was an important personalisation features discussed in the workshops and rated in the community survey. A multi-pronged approach that included both online and in-person features, such as links to community events, was suggested in the workshops and indirectly supported in the community survey when respondents indicated support for local community markets and events.

### Divergent themes

The inclusion of cooking resources, such as videos, was well supported in the community and research partner workshops. However, this resource was the least supported feature in the community survey. A kids’ section with interactive games was suggested in the workshops, however feedback from the community survey indicated that few respondents would find this useful, and many indicated that they would never use interactive games to get family and friends involved.

### Prioritisation of digital intervention functions

**Fig. 2:**
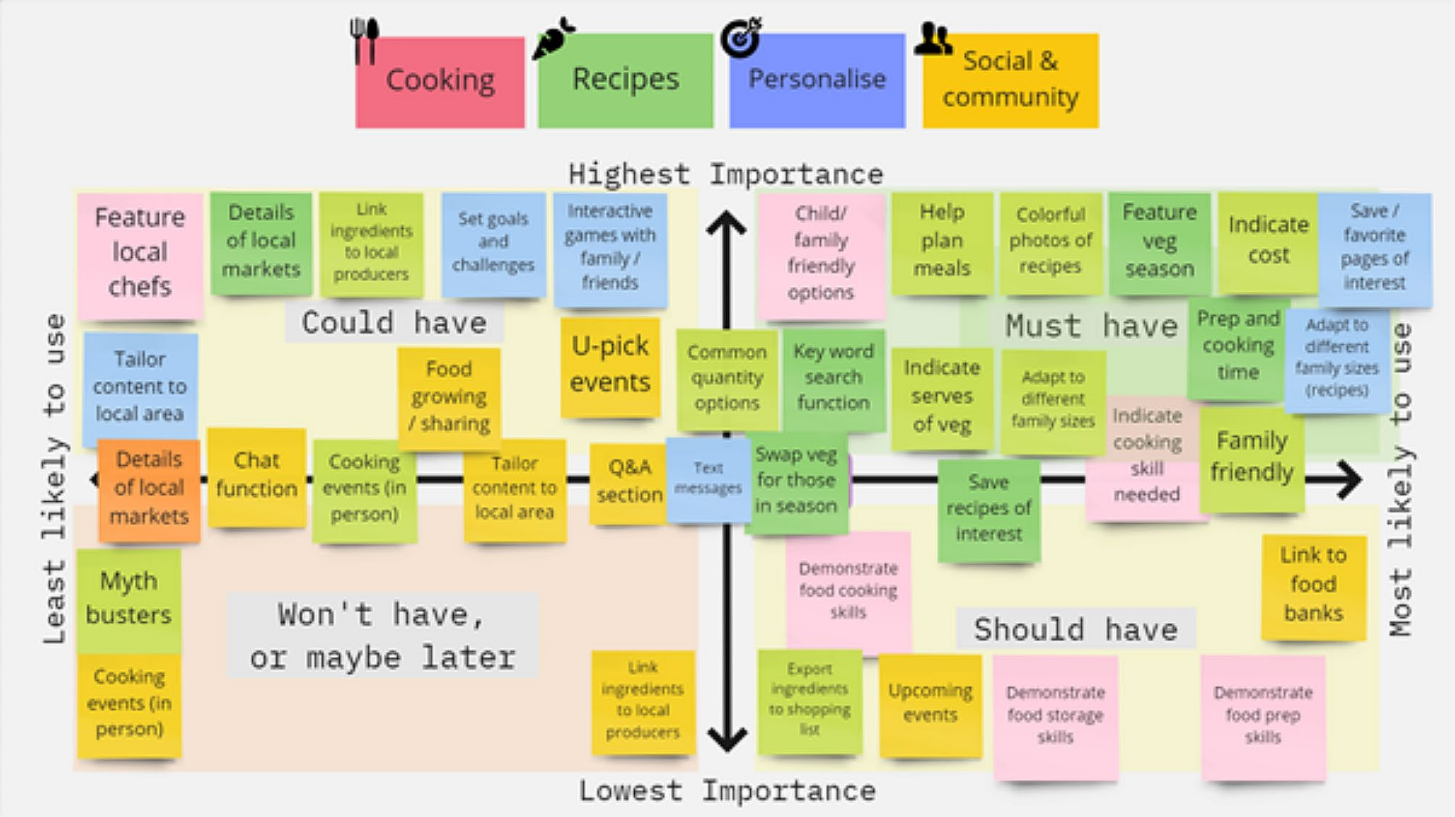
MoSCoW prioritisation of functions of the digital intervention identified by participants in the final co-design workshop

As shown in Fig. 2, the MoSCoW prioritisation conducted in workshop 8 identified the ‘Must have, Could have, Should have or Won’t have or maybe later’ functions of the following digital intervention features: recipe, social and community, personalisation and cooking resources. Many recipe functions and some personalisation features were ranked as ‘must haves’. Cooking resources received a mixed response; the functions were ranked as ‘must, should and could have’. Most of the social and community features were ranked ‘won’t have or maybe later’, with the exception of food banks and local events, which were ‘should haves’.

I think maybe as the website or the app grows, it could be something that you could add as more people in local areas start to use it. So you know how some websites have a FAQ section or something? There could be a little link under there where you could choose your area… but…not over complicated because it could detract from the really important things that you would want to spend more time on. (Community participant, female aged 33, South West region, Western Australia)

When discussing the social and community features, food banks were positively viewed by participants, however the limited offerings of fresh and frozen vegetables was noted as a challenge.

Food banks depend massively on what donations they receive, and they seem to receive a lot of long-life things like biscuits and chips. And where I am, there’s very, very rarely even frozen fruit and vegetables, let alone fresh fruit and vegetables. It’s primarily potatoes, bread, and your long-life shelf things. (Community participant, female aged 33, South West region, Western Australia)

Further, much of the discussion related to feasibility of implementing such features, for example, who would deliver local events across large rural regions.

Council areas are so huge, then you’ve got all of these smaller sections and farmers, and local people at the local level so I think that will be a very difficult one to implement as well. (Community participant, female aged 58, Loddon Campaspe Victoria)

When consolidated into a list of co-design findings, several implementation recommendations emerged to address individual and environment level barriers to vegetable intake in rural communities (Table 3). These recommendations included improving food literacy, utilising personalisation and goal-setting options to improve retention and encouraging social connection by accessing local initiatives available in their food environments.

**Table 3:** Mapping priority intervention features to the NOURISHING framework and implementation recommendations

Co-design findings			Implementation recommendation	
Theme	NOURISHING Domain/ Policy area	Participant quote	Topic	Recommendation
Recipes and cooking	BCC/ Education and skills	“Recipes are always good because there’s so many people that are lost. […] Like yesterday, I picked up eggplants, they look wonderful, and I said, “What can I do with this?”	**Food literacy**: address barriers to preparing and cooking vegetables by providing access to vegetable-rich recipes and healthy eating advice.	Mechanisms to improve food literacy across all population groups are required.
Personalisation	BCC/ Nutrition advice	“Yeah, so you can set the reminder yourself for what you want. Have you done the shopping, or if you’re pre-planning your shopping and your meal, so it will just give you a reminder then.”	**Personalisation and goal-setting**: address barriers to retaining engagement by offering ways to customise and tailor content to the needs and preferences of the individual.	Mechanisms to integrate data at scale are required.
Community	Food environment/ Offer healthy foods	“Sharing food is important because we’d also build up relationships in the community.”	**Social connection**: address barriers to accessing vegetables by providing information on local rural food initiatives.	Mechanisms to maintain the accuracy of online information at the local food environment-level are required.

The NOURISHING framework was used to identify Domains and Policy Areas [27]; BCC, Behaviour change communication

## Discussion

This paper describes the co-design of a digital intervention to improve vegetable intake. Co-design was undertaken across three levels, with adults living in rural communities and research partners representing three local rural government and one peak non-government health organisation. An iterative co-design process was adopted to understand the needs (pre-design phase) and ideas (generative phase) of the target populations. Consensus was reached using prioritisation methods (evaluative phase), resulting in features and functions for a digital intervention that address individual and food environment barriers to vegetable intake. Implementation recommendations centred on (i) food literacy approaches to improve skills via access to vegetable-rich recipes and healthy eating resources, (ii) access to personalisation options and behaviour change support and (iii) improving the community food environment by providing information on, and access to, local food initiatives. The next steps for this work are to describe the prototyping (evaluation phase) and feasibility testing (post-design phase) of this intervention.

The most prominent focus of the workshops was on approaches to support increased vegetable intake addressing food literacy. Although multiple definitions and frameworks have been proposed [29, 30], food literacy is broadly defined as proficiency in food-related skills and knowledge [31]. The focus on skills and knowledge was reflected in workshop discussions on the provision of recipes and cooking videos, where participants valued visually appealing, quick-to-access and easy-to-use recipes, to provide them with ideas and skills to incorporate more vegetables into meals. Research suggests that increasing the variety of vegetables consumed leads to increased intake overall [32]. Therefore, to support self-efficacy in purchasing and preparing a variety of vegetables, food literacy resources should ensure that recipes and educational resources include a variety of vegetable types. Such approaches are warranted in this population group since mean serves of vegetables were as low as 1.4 serves/day, 3.6 serves lower than recommended and 1.7 serves lower than average national intakes among adults [33]. While, the personalisation of recipes to local and seasonal produce emerged from the co-design workshops as a concept unique to rural communities, the use of recipes is consistent with the focus of many previous digital interventions to increase vegetable intake [16]. For example, the VegEze mobile app utilised education (> 50 recipes and meal suggestions) and motivation (goal-setting and challenges) approaches to increase vegetable intake and variety in 1224 Australian adults [34]. However, engagement rapidly declined over the 90-day intervention, suggesting that education and motivation alone may not be sufficient to maintain initial effects.

A strong theme throughout the workshops was the importance of community food environments and social connection in rural communities. Consistent with literature on rural communities [35, 36], access to healthy foods via food sharing and growing initiatives were positively viewed by participants, and seen as opportunities to strengthen community values and food security. This included the use of food banks, which are critical for addressing food security in times of crisis, but often lack consistency in the nutritional quality of food available and an ability to accommodate culturally specific dietary needs and preferences [37]. However, to date, few digital interventions aiming to increase vegetable intake have incorporated features that address barriers at both the individual- and food environment-level [16]. This may be due to challenges associated with setting-based interventions being delivered digitally, which also arose in the present workshops. For example, despite the strong desire for integration of these features and functions into the digital intervention, prioritisation led to most of the social and community features (such as links to local producers) being ranked as ‘won’t have or maybe later’. This ranking reflected discussions on concerns about feasibility in relation to whether online information on local food resources would be up-to-date. Nonetheless, local events and foodbanks were prioritised as ‘should haves’, reflecting the importance of such initiatives irrespective of feasibility concerns. As a result, implementation recommendations for this theme include establishing mechanisms to maintain the accuracy of online information at the local food environment-level. For example, with the increasing digitalisation of food environments and high adoption of digital tools in young adult populations [38], embedding online data from Google or food retailers, could help facilitate this [39].

The use of the Evaluating Nonadoption, Abandonment, and Challenges to the Scale-Up, Spread, and Sustainability of Health and Care Technologies (NASSS) framework [40] may aid in ensuring effective implementation, scale up and roll out of this digital intervention. This can be achieved by being explicit in identifying and addressing challenges across the 7 NASSS domains: the condition, technology, value proposition, adopter system, health or care organization(s), the wider (institutional and societal) context and embedding and adaptation over time.

A final implementation recommendation that arose from the workshops was the personalisation of the digital intervention to meet the needs of the individual. This is a commonly used approach, adopted by over 75% of existing digital interventions to increase vegetable intake [16]. For example, in a 6-month text message-delivered intervention, participants had the opportunity to customise their degree of personalisation, based on preferred frequency and timing of text messaging. In the present study, participants, particularly young adults, suggested that reminders would be helpful for self-monitoring their goals to shop, prepare and consume, more vegetables. Customisation of the digital intervention was also positively viewed by participants and identified as priority functions, where favouriting and filtering information were identified as helping to ensure the intervention material was relevant and engaging. This is consistent with a recent study of the personalisation of digital health information, where young adults preferred user-driven personalisation to be able to customise their experience, as distinct from system-driven personalisation [41]. 

The present study has several strengths and limitations. The iterative workshop design was a strength as it allowed participants to explore, conceptualise and refine ideas in a true co-design manner. However, this may have created barriers to recruitment and may have limited our sample size, as participants were required to attend on multiple occasions over 2 months. Further, young adults were under-represented, and due to challenges in finding a time that suited all participants, two additional workshops had to be conducted rather than one. This limited the sharing and refining of young adults’ ideas with other participants, which may have contributed to the divergent themes observed despite them being coded by age group. Although the small sample size and particular geographic region included in this study limits generalisability to the wider rural Australian population, the resulting intervention will first test the feasibility in the same geographic region prior to testing in a larger and more representative sample. Thus, this is an appropriate first step. Further, although changes in staffing of the research partners was a limitation, all new partners were briefed on the project beforehand. It is possible that exemplars selected by researchers to present to participants to stimulate discussion may have shaped the findings. Nonetheless, many new ideas emerged from participants. Lastly, the online and interactive delivery of the workshops was a strength as it bridged geographical gaps, reduced participant burden associated with travel time, and aligned with COVID-19 restrictions, whilst still providing tools for participants to use to feel engaged.

## Implications for future research

This paper highlights the importance of co-design with the intended audience as it resulted in both individual-level and local food environment-level features of the interventions that are different to previous digital vegetable interventions. Future research is needed to see if these features are acceptable and well used (feasible), if they are important in driving behaviour change (efficacy) and whether the intervention can be easily adopted and sustained (implementation potential). The first of these research needs is being addressed in the Veg4Me study [42]. These activities will continue to engage stakeholders in the evaluation and post-design phases of the co-design process. Future research should also explore opportunities to engage greater gender diversity in the co-design process. As this study is the first to describe the co-design of a digital intervention to increase vegetable intake in adults living in rural communities, a greater body of research investment is required to address the diet and health inequities experienced by rural communities. The increasing number of co-designed digital interventions may help ensure that dietary interventions are more effective in addressing the growing burden of chronic disease.

## Conclusions

Through an iterative co-design process, rural-dwelling adults expressed preferences for access to vegetable-rich recipes and healthy eating resources, personalisation options and behaviour change support and information on, and access to, local food initiatives. When priority features were mapped to the NOURISHING framework and implementation recommendations, these features spanned domains of behaviour change communication and the food environment and addressed needs for improved food literacy, personalisation/ goal-setting and social connection. Conclusions regarding its feasibility, efficacy and implementation potential cannot yet be determined. Further work is needed to engage stakeholders in the development of prototypes and in the creation of a final digital intervention for testing in rural communities. Future research should ensure the use of iterative co-design processes in developing digital dietary interventions.

### Electronic supplementary material

Below is the link to the electronic supplementary material.

Supplementary Material 1

## Data Availability

The datasets generated during the current study are not publicly available due to ethics requirements but are available from the corresponding author on reasonable request.

## Abbreviations

COREQ: Consolidated Criteria for Reporting Qualitative Research
MoSCoW: Must-have, Should-have, Could-have, and Won’t-have or will not have right now
NAAAS: Evaluating Nonadoption, Abandonment, and Challenges to the Scale-Up, Spread, and Sustainability of Health and Care Technologies
SEIFA: Socio-Economic Indexes for Areas
