# Supplementary material for: Co-design of a personalised digital intervention to improve vegetable intake in adults living in Australian rural communities
Source: BMC Public Health. 2024 Jan 10;24:146. doi: 10.1186/s12889-024-17641-8 (PMC10782626; doi:10.1186/s12889-024-17641-8)
Supplement: Supplementary file 1 — Supplementary Material 1 [file 12889_2024_17641_MOESM1_ESM.docx]

**Supplemental Table 1.** COREQ (COnsolidated criteria for REporting Qualitative research) Checklist

| **Topic** | **Item No.** | **Guide Questions/Description** | **Page No.** |
| --- | --- | --- | --- |
| **Domain 1: Research team and reflexivity** | | |  |
| *Personal characteristics* | | |  |
| Interviewer/facilitator | 1 | Which author/s conducted the interview or focus group? | 10 |
| Credentials | 2 | What were the researcher’s credentials? E.g. PhD, MD | 10 |
| Occupation | 3 | What was their occupation at the time of the study? | 10 |
| Gender | 4 | Was the researcher male or female? | 10 |
| Experience and training | 5 | What experience or training did the researcher have? | 10 |
| *Relationship with participants* | | |  |
| Relationship established | 6 | Was a relationship established prior to study commencement? | 10 |
| Participant knowledge of the interviewer | 7 | What did the participants know about the researcher? e.g. personal goals, reasons for doing the research | 10 |
| Interviewer characteristics | 8 | What characteristics were reported about the inter viewer/facilitator? e.g. Bias, assumptions, reasons and interests in the research topic | 10 |
| **Domain 2: Study design** | | |  |
| *Theoretical framework* | | |  |
| Methodological orientation and Theory | 9 | What methodological orientation was stated to underpin the study? e.g. grounded theory, discourse analysis, ethnography, phenomenology, content analysis | 6 |
| *Participant selection* | | |  |
| Sampling | 10 | How were participants selected? e.g. purposive, convenience, consecutive, snowball | 6-12 |
| Method of approach | 11 | How were participants approached? e.g. face-to-face, telephone, mail, email | 6-12 |
| Sample size | 12 | How many participants were in the study? | 6-12 |
| Non-participation | 13 | How many people refused to participate or dropped out? Reasons? | 6-12 |
| *Setting* | | |  |
| Setting of data collection | 14 | Where was the data collected? e.g. home, clinic, workplace | 6-12 |
| Presence of non-participants | 15 | Was anyone else present besides the participants and researchers? | 6-12 |
| Description of sample | 16 | What are the important characteristics of the sample? e.g. demographic data, date | 6-12 |
| *Data collection* | | |  |
| Interview guide | 17 | Were questions, prompts, guides provided by the authors? Was it pilot tested? | 6-12 |
| Repeat interviews | 18 | Were repeat interviews carried out? If yes, how many? | 6-12 |
| Audio/visual recording | 19 | Did the research use audio or visual recording to collect the data? | 6-12 |
| Field notes | 20 | Were field notes made during and/or after the interview or focus group? | 6-12 |
| Duration | 21 | What was the duration of the interviews or focus group? | 6-12 |
| Data saturation | 22 | Was data saturation discussed? | 6-12 |
| Transcripts returned | 23 | Were transcripts returned to participants for comment and/or | 6-12 |
| **Domain 3: analysis and findings** |  |  |  |
| *Data analysis* |  |  |  |
| Number of data coders | 24 | How many data coders coded the data? | 16 |
| Description of the coding tree | 25 | Did authors provide a description of the coding tree? | 16 |
| Derivation of themes | 26 | Were themes identified in advance or derived from the data? | 16 |
| Software | 27 | What software, if applicable, was used to manage the data? | 16 |
| Participant checking | 28 | Did participants provide feedback on the findings? | 16 |
| *Reporting* |  |  |  |
| Quotations presented | 29 | Were participant quotations presented to illustrate the themes / findings? Was each quotation identified? e.g. participant number | 16 |
| Data and findings consistent | 30 | Was there consistency between the data presented and the findings? | 17-22 |
| Clarity of major themes | 31 | Were major themes clearly presented in the findings? | 17-22 |
| Clarity of minor themes | 32 | Is there a description of diverse cases or discussion of minor themes? | 17-22 |

**Supplemental Table 2**. Co-design workshops and community survey

| Workshops/ community survey | Key activities/components |
| --- | --- |
| Workshop 1 – Pre-design  (Community members) | - Ice breaker activity, i.e. discussion of barriers to vegetable intake. - Participants commented on three exemplar online tool inclusions (i.e., recipes; goal-setting; food sharing). - Participants divided into two groups to brainstorm aspirational tool ideas. |
| Workshop 2 – Pre-design  (Research partners) | - Icebreaker activities repeated from workshop 1: - Discussion of barriers to vegetable intake. - Participants commented on three exemplar online tool inclusions (i.e., recipes; goal-setting; food sharing). - Participants divided into two groups to brainstorm aspirational tool ideas. |
| Workshop 3 – Generative  (Community members) | - Participants commented on workshop 1 and 2 summary. - Participants asked to report on likes/dislikes about exemplar meal planners, recipes, cooking tips and videos from the National Heart Foundation. - Participants commented on an example of a social marketing campaign and ideas about how to work with local food retailers/environments. - Participants divided into two groups and brainstormed further ideas for what a tool could look like. |
| Workshop 4 – Generative  (Research partners) | - Participants presented with the summary from workshop 3. - Participants presented with the same examples shared in workshop 3, incorporating workshop 3 participant comments. - Participants commented on exemplars and discussed ideas for what an online vegetable consumption tool could look like. |
| Workshop 5 – Generative  (Community members) | - Participants presented with a summary of the main discussions from workshops 3 and 4. - Participants presented with proposed key elements of an online tool, informed by workshops 1–4, and asked to provide additional feedback on these elements. - Workshop participants commented on anticipated acceptability of the tool, and suggested tool names. |
| Workshops 6 and 7 – Generative  (Community members) | - Ice breaker activities: thinking about barriers to vegetable intake; discussion about previous experiences of using online food-based apps or websites. - Presentation and discussion about four online examples: 1) recipes; 2) cooking videos and tips; 3) setting and tracking goals; and 4) food sharing. - Participants asked to brainstorm ideas for what an online tool could look like. - Discussion about additional supports to help engage tool users. - Discussion of findings from previous five workshops. |
| Community survey – Generative / Evaluative  (Community members) | - Open- and close-ended questions on the main features and functions of a tool suggested in workshops 1–6. |
| Workshop 8 – Evaluative (Community members and research partners) | - MoSCoW (‘Must-have, Should-have, Could-have, and Won’t-have or will not have right now’) prioritisation methodology to collectively rank features of the online tool arising from the co-design workshops and community survey. |

Workshops are mapped to the phases of co-design (pre-design, generative, evaluate and post-design)
